# Providing an Outdoor Exercise Area Affects Tie-Stall Cow Reactivity and Human-Cow Relations

**DOI:** 10.3389/fvets.2020.597607

**Published:** 2021-01-12

**Authors:** Nadège Aigueperse, Elsa Vasseur

**Affiliations:** ^1^University of Clermont Auvergne, INRAE, VetAgro Sup, UMR Herbivores, Saint-Genès-Champanelle, France; ^2^Department of Animal Science, McGill University, Ste-Anne-de-Bellevue, QC, Canada

**Keywords:** dairy cows, behavioral robustness, well-being, emotions, handling

## Abstract

Confinement and restriction of movement are a reality for most dairy cows. Providing outdoor access is one method to increase movement opportunities. However, leading cows to an outdoor exercise area increases their exposure to manipulations different from those of an indoor housing system. These situations have the potential to induce fear reactions, which can lead to injuries for the cow and danger or economic losses for the farmer. Our aim was to evaluate the development of the human-cow relationship and general reactivity of cows after a 12-week period of outdoor access provision in winter, summer and fall. A total of 16 cows in the winter, 16 in the summer, and 15 in the fall were enrolled in the study and either allocated to the treatment (Out) or stayed in the tiestall (NonOut). A human reactivity test and suddenness test were performed before and after the 12-week treatment period. In winter and to a lesser extent in fall, Out cows had a better human reaction score compared to NonOut cows, suggesting that cows with outdoor access during the winter associated human approaches with positive events. Conversely, no difference in the human reaction score was found between treatments during the summer. For summer and fall, Out cows did, however, show a decrease in their reaction score to the suddenness test compared to NonOut cows. The results of the human reactivity test in the summer suggested that cows with outdoor access did not associate the manipulation with a positive event. Interestingly, this result is not due to the cows being more frightened, since the suddenness test suggested that the Out cows were less fearful than NonOut cows. The way in which cows were led to the outdoor area could explain the differences in cow responses. Here, summer cows faced greater movement restrictions during trips to the outdoor area than in the winter, which may have been negatively perceived by the cows. We conclude that, besides the provision of outdoor access, the manner in which cows are handled during these events may have significant impacts on their reactions and could facilitate future handling.

## Introduction

Farm animals are often selected for their high production capacities. On occasion, behavioral characteristics can generate deficits or problems of adaptation (1, 2). The intensification of dairy production has led to changes in the management and housing parameters for animals, which now require new adaptations from them. Farm animals, particularly in intensive milking systems, can be subjected to many manipulations, which they are not accustomed to or could be considered as aversive, such as for certain medical interventions. These manipulations can cause significant stress in the animal (3). In times of stress, the animal's reaction can be variable, unpredictable, and dangerous both for them and their handlers (4–7). A balanced emotional reaction will allow the animal to respond to a potentially dangerous stimulus without becoming overwhelmed. This will prevent animals from over-reacting to novel situations, particularly during handling, and thus reduce injury risk for themselves and their handler.

Handling is an important risk in animal farming. Although cows are relatively docile animals, they can be dangerous and aggressive when situations displease or frighten them (6). Previous situations that involve discomfort have been associated with difficult handling. For example, moving cows for hoof-trimming results in more fearful and aggressive behaviors compared to directing them to daily milking (7). Numerous studies have shown that an animal's early experiences during handling strongly affect their future responses (8, 9) and more generally, the human-animal relationship (10). Other studies have shown a direct link between human behavior and cow behavior (11), where aversive manipulations can impair the relationship with humans [for review: (12)]. For example, Breuer et al. (13) found that heifers that were negatively handled had a greater flight distance toward humans and were more stressed than positively handled cows.

The provision of exercise is recommended to improve animal well-being and foot health (14, 15), and increase behavioral opportunities [review by (16)]. For tie-stall cows, this implies introducing many stimuli and additional human manipulations that they are not used to. Cows may be afraid and react unpredictably to these situations, or even aggressively, which may impact animal and human welfare. Providing stimulation can also help the animal to respond in a more appropriate way and therefore adapt better to situations (17, 18).

The objective of this research was to study the impact of regular exercise provision in an outdoor area, in winter, fall and summer seasons, on the reactivity and relationship of tie-stall cows with humans. Two behavioral tests were carried out: a human relationship test and a suddenness test for reactivity. Our hypothesis predicted that the daily manipulation of animals, combined with an positive outing experience, would improve the human-animal relationship. In addition, the enrichment provided by access to an outdoor exercise area may help the animal become less reactive to sudden events by building more adapted behaviors.

## Materials and Methods

### Animal and Housing Conditions

Forty tie-stall housed Holstein dairy cows were selected from the resident herd at the McGill University Macdonald Campus Dairy Complex (QC, Canada). During the study, cows were housed in a tie-stall barn consisting of cubicle tie stalls (stall width of 1.3 m, bed length of 1.9 m, stall length of 2.1 m) with rubbermats, a 2 cm depth of wood shavings for bedding, and concrete alleyways. Cows within a pair were positioned to alternate in adjacent stalls. They had access to water *ad libitum*, and feed rations (average of 21.1 kg/d of TMR comprised 48.0% hay, 46.7% silage, 4.3% protein supplement, and 1.0% vitamin and mineral supplement) were distributed 4 times per day to ensure that feed was always available. Cleaning of the alleyways and stalls occurred 4 times per day, in equal intervals before and after outings. Fresh bedding was provided as needed to maintain a 2 cm depth of wood shavings per stall. The outdoor exercise area was a pen delimited by electric wires within a grassland (300 m^2^). In winter, there were snow cover and wood chips and in summer/fall, the grass was cut. The surface per cow averaged 25 m^2^ across seasons. The size of pens varied between and within seasons and each group of cows was allocated to a new pen each week. In winter and fall, cows were allocated to pens of different sizes across the trial (min-max: 10-40 m^2^ per animal); in the fall, the size of the paddock was always the same (39 m^2^ per animal). An alleyway going from the barn to each pen allowed the handler to move the animals to their respective pens.

### Procedures

#### General Process

Enrolled cows (excluding companion cows) were randomly allocated to 3 seasons, for a total of 16 in the winter, 16 in the summer, and 15 in the fall. This study was part of a series of trials examining the effects of exercise access in tie-stall cows and the number of animals enrolled was chosen according to several objectives (most notably: cow locomotor activity). Within each season, the cows were randomly assigned to one of two groups (Out or NoOut), and balanced and paired by parity and stage of lactation (Parity: 2.2 ± 1.22; DIM: 140.7 ± 71.12). Treatment cows (Out) were taken outdoors to an exercise area, while control cows (NonOut) were kept in the tie-stall. Outings took place 5 days a week each morning during 12 weeks. If some cows were in heat, they were not led outside to avoid injuries related to their excitement. Out cows were taken outdoor along with one not tested companion (Winter, Summer) or with the two other cows (Fall). This was done to ensure that all cows could have at least one conspecific with which to engage in social interactions. With all “Out” groups outside in the exercise yard, there was a total of 16 (Winter, Summer) or 15 (Fall) cows in the exercise yard at once (including trial and companion cows). All groups were put in separate paddocks. Out cows with their companion cow(s) were taken outdoors, pair by pair (or in trio), by being untied and equipped with a halter and moved to their outdoor exercise area for 2 to 3 h each morning. When released from the tie-stall, cows were halter-led by a handler through the barn until the outdoor. Then, the handler let go of the halter and moved the cows through an outdoor walking corridor, and finally directed them toward an outdoor exercise enclosure. Handling was more restrictive in summer than in fall, and in fall than in winter trials, partly due to changes in the flooring conditions of the alleys leading to the outdoor yard: cows moved forward differently according the floor stability and the weather. Handling was carried out according to a pre-established and standardized protocol to ensure the most consistent handling possible between cows, and was adapted for each season (Supplementary Material S1).

Two behavioral tests were performed: a human test and a suddenness test. These tests were carried out on all cows before and after the 12 weeks treatment period (Table 1). Not all cows could be tested at all times, due to estrus on testing days (baseline), or due to a lack of treatment application or a combination of events including estrus, weather, and health conditions (after treatment application period).

**Table 1:** Numbers of tested cows by treatment, phase and seasons.

	**Winter**		**Summer**		**Fall**	
**Phase**	**Before**	**After**	**Before**	**After**	**Before**	**After**
N NonOut	8	7	8	8	9	9
N Out	6	8	8	7	6	6

Cows were randomly subjected to the two behavioral tests on three consecutive days with not more than one test per day. The same test was not carried out on two neighboring cows in a single day, and the tests were equally distributed across groups each day. The suddenness test, which can be disruptive to other cows in proximity to the test cow, was always done after the human test.

### Behavioral Tests

#### Human Test

The test is adapted from the procedure by Herskin et al. (19) and similar to Schmied et al. (20). The test involved two individuals: a test person who is used as stimulus, and an observer. The test person was an unfamiliar female dressed in blue work coat, different from the one that takes out the animals. She was the same for the winter and summer seasons, but was different in fall for technical reasons. However, the stature and clothes were noticeably similar. To begin, the observer ensured that all cows were standing. If they were lying down, she clicked her tongue then gave a little push on the rump if necessary; this method being usually and very regularly used by farm members throughout the day. The observer then positioned herself at the end of the row, at least 2 stalls away from the target cow, and performed live scoring. After waiting 5 min, in order to avoid any influence of the forced standing up, the test person stood in front of the target cow at a distance of 1.30 m from the tierail and captured the test cow's attention but to avoid a stretched chain at the start of the test (see Video S1 in Supplementary material). When she was ready, the observer started the timer and then, the test person approached the test cow every 5 s according to the following sequence:

- Stage 1: 1 step, arms placed alongside the body
- Stage 2: 1 step, arms placed alongside the body
- Stage 3: One arm stretched out at ~45°
- Stage 4: Outstretched hand placed on the chain at the base of the neck

The observer noted the reaction of the cow at each stage according to the following numerical scores:

- Score−3: Steps back (steps >2), chain stretched to the maximum,
- Score−2: Steps backs (1 or 2 steps)/struggles (for stage 4)
- Score−1: Turns the head back or away
- Score 0: Looks at the person
- Score +1: Approaches the person without touching, sniffs
- Score +2: Approaches and touches the person
- Score +3: Tries to lick/catch the person with the mouth (clothing or hand with the tongue or the lips).

This test was repeated 3 times per cow, with a rest period of at least 5 min between each test period. The mean of each score by stage and by cow was calculated. For example, a frightened cow could score the first time: −2, −2, −3, −2; the second time −1, −2, −3, −3; and the third time: −2, −1, −2, −3: so for each stage the mean for this cow would be: −1.67, −1.67, −2.67, −2.67. For a calm cow, it would be: 0, 0, −1, −1; 0, 0, 1, −1; 0, 0, 0, 0; so the mean would be: 0, 0, 0, −0.67.

#### Suddenness Test

In winter trial, we tried another test that finally could not be implemented for technical reason; therefore, data were only collected in summer and fall. The aim of this test was to evaluate the reactivity of the cow to a sudden event. Therefore, we dropped an object in front of the target cow and noted the reaction. A white plastic bowling pin (H = 45 cm ø =10 cm) hanging on a fine string was used as a stimulus. The previous evening, the bowling pin were installed above the cows, out of their reach and field of vision. All cows were in a standing position 5 min before the test. The video recording (GoPro®, San Mateo, USA) was started 1.5 min before the start of the test for a 2-min duration, using a camera mounted on tripod in front of the cows (cows were previously habituated to the procedure). After 1.5 min, the manipulator dropped the bowling pin by releasing the string suddenly. He remained approximately two stalls away from the target cow not to disturb the cow. The recording then continued for 30 s (see Video S2 in Supplementary material).

Using the videos, an observer noted the duration of freezing expressed by the cows (time spent freezing in fixation on the object). The cows were also assigned a reaction score from 0 to 4 according to the following behaviors: No reaction (0); startled, with no backward movement (1); startled, with backward movement of 1 or 2 steps (2); startled, with strong backward movement or taut chain (3); startled, with strong backward movement (with struggle) and taut chain (4).

### Statistical Analysis

Winter, summer and fall trials were not designed as replicates but as independent trials to account for large differences in climatic conditions, age of animals, restrictiveness of animal handling, staff availabilities, and responsiveness of animals to flooring conditions and handling methods which were different between trials. Each season was analyzed and reported separately across the manuscript.

We checked the homogeneity of the variance by a Levene test. All data, except the sudden reaction score, was determined to be normally distributed assessed graphically using Q-Q Plot. We implemented a linear mixed-effects model for all scores of human test and for freezing duration in the suddenness test. For the suddenness tests, we implemented a cumulative link mixed-effects model with the reaction score as ordinal variable. For each model, we have considered the following factors:

1. Phase as a fixed effect: before or after 12-week exercise period
2. Treatment as fixed effect: Out cows with exercise or NonOut cows without exercise
3. Phase x Treatment interactions as a fixed effect
4. Animal nested in pair (pairs formed according to parity and stage of lactation) as a random effect.

Residual normality was visually assessed using a Q-Q plot. *Post-hoc* comparisons were performed by least significant difference (LSD) tests. The threshold of significance was 0.05, and tendencies between 0.1 and 0.05 are mentioned. For the results from the 4 stages of the human test, a Bonferroni correction was applied for multiple comparisons so the threshold of significance considered is 0.05/4 = 0.0125 and 0.025 for tendencies. Statistical analyses were performed in SPSS 20.0 (®IBM, SPSS Inc., Chicago, Illinois, USA).

## Results

### Human Test

In winter, the first difference occurred at the third stage with a phase^*^treatment effect (*F*_3,26_ = 7.82; *P* = 0.012): after the outdoor exercise access period, cows without exercise (NonOut) had the lowest score on the human test (Table 2). In the fourth and last stage, we had a phase^*^treatment effect (*F*_3,26_ = 9.93; *P* = 0.004): after the outdoor exercise access period, Out cows had a higher score compared to before the outdoor exercise access period (LSD, *P* = 0.004), and were less avoidant than NonOut cows (LSD: *P* = 0.004).

**Table 2:** Estimated mean (± S.E.) of cow reactivity scores for each stage of human approach.

**Season**	**Phase**	**Treatment**	**Stage 1**	**Stage 2**	**Stage 3**	**Stage 4**
Winter	Before	Out	−0.23 ± 0.33	−0.37 ± 0.55	0.11 ± 0.57	−1.21 ± 0.60 **a**
		NonOut	0.22 ± 0.28	0.19 ± 0.47	0.56 ± 0.49	−1.22 ± 0.51 **a**
	After	Out	0.08 ± 0.44	0.34 ± 0.45	0.83 ± 0.38	0.21 ± 0.45 **b**
		NonOut	−0.28 ± 0.43	0.25 ± 0.45	−0.36 ± 0.37	−1.76 ± 0.44 **a**
Fall	Before	Out	−0.33 ± 0.32	0.27 ± 0.57	−1.07 ± 0.36 **a**	−1.67 ± 0.57 **a**
		NonOut	−0.34 ± 0.24	0.11 ± 0.42	−0.63 ± 0.27 **ab**	−1.15 ± 0.42 **ab**
	After	Out	0.07 ± 0.33	0.40 ± 0.51	0.33 ± 0.49 **b**	−0.27 ± 0.52 **b**
		NonOut	0.04 ± 0.24	0.19 ± 0.38	−0.11 ± 0.37 **ab**	−0.78 ± 0.39 **ab**
Summer	Before	Out	−0.38 ± 0.23	−0.49 ± 0.33	−0.07 ± 0.31	−1.88 ± 0.31
		NonOut	−0.08 ± 0.23	−0.21 ± 0.33	−0.38 ± 0.31	−1.63 ± 0.31
	After	Out	−0.39 ± 0.30	−0.04 ± 0.52	−0.72 ± 0.49	−1.10 ± 0.45
		NonOut	−0.71 ± 0.28	−0.71 ± 0.50	−0.58 ± 0.47	−1.63 ± 0.42

*Values are displayed separately before and after an outside exercise period (Out) vs. remaining in tie-stalls without an exercise period (NonOut) for each season. Stage 1 represents the most distant position while stage 4 was the most intrusive (when the chain was caught at the neck)*.

*Data represents mean ± S.E. Different letters indicate differences between groups. Post-hoc LSD tests with Bonferroni corrections (significance with P < 0.0125)*.

In fall, the first difference occurred at the third stage with a phase effect (*F*_1,26_ = 10.22; *P* = 0.004). After the outdoor access period, Out cows had a higher score on the human test compared to before the application of the treatment (LSD: Stage 3: *P* = 0.008; Stage 4: *P* = 0.002; Table 2), demonstrating that they approached more and were less avoidant of human stimuli after regular outings.

In the summer, there were no differences between the cows, nor in terms of treatment, phase, or treatment^*^phase interaction (*P* > 0.05 for all; Table 2).

### Suddenness Test

**Figure 1:**
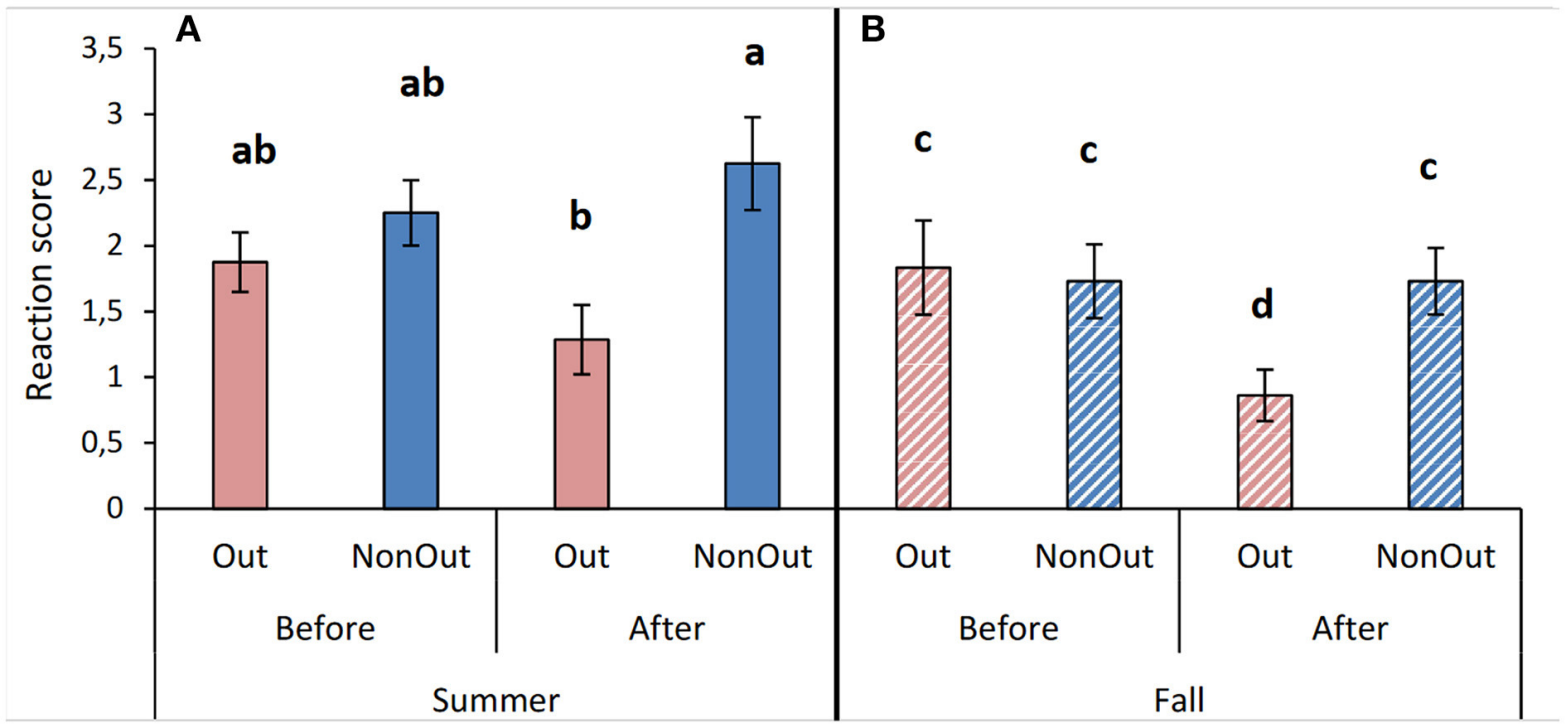
Mean (± S.E.) of cow reaction scores for the suddenness test, measured after dropping an object in front of the cows. Values are displayed before and after an outside exercise period (Out) vs. remaining in tie-stalls without an exercise period (NonOut), for the summer **(A)** and fall seasons **(B)**. Means with the same letter in the same part of the column are not significantly different (*P* > 0.05).

In summer, the cows' reactions to a fallen object tended to be different according to treatment^*^phase (*F*_3,25_ = 3.21; *P* = 0.09). *Post-hoc* comparisons showed that after the outdoor exercise access period, Out cows had a weaker reaction to the fall of the object than NonOut cows (LSD: *P* = 0.0001, Figure 1A) and tended to have a weaker reaction than before (LSD: *P* = 0.053). In the fall, a difference was observed between the cows' reactions to a fallen object according to treatment^*^phase (*F*_3,24_ = 4.52; *P* = 0.044). *Post-hoc* comparisons showed that after the outdoor exercise access period, Out cows had a weaker reaction to the fall of the object than Out cows and NonOut cows before the outdoor exercise access period (LSD: *P* = 0.001), and weaker than NonOut cows after the outdoor exercise access period (LSD: *P* = 0.023, Figure 1B).

**Figure 2:**
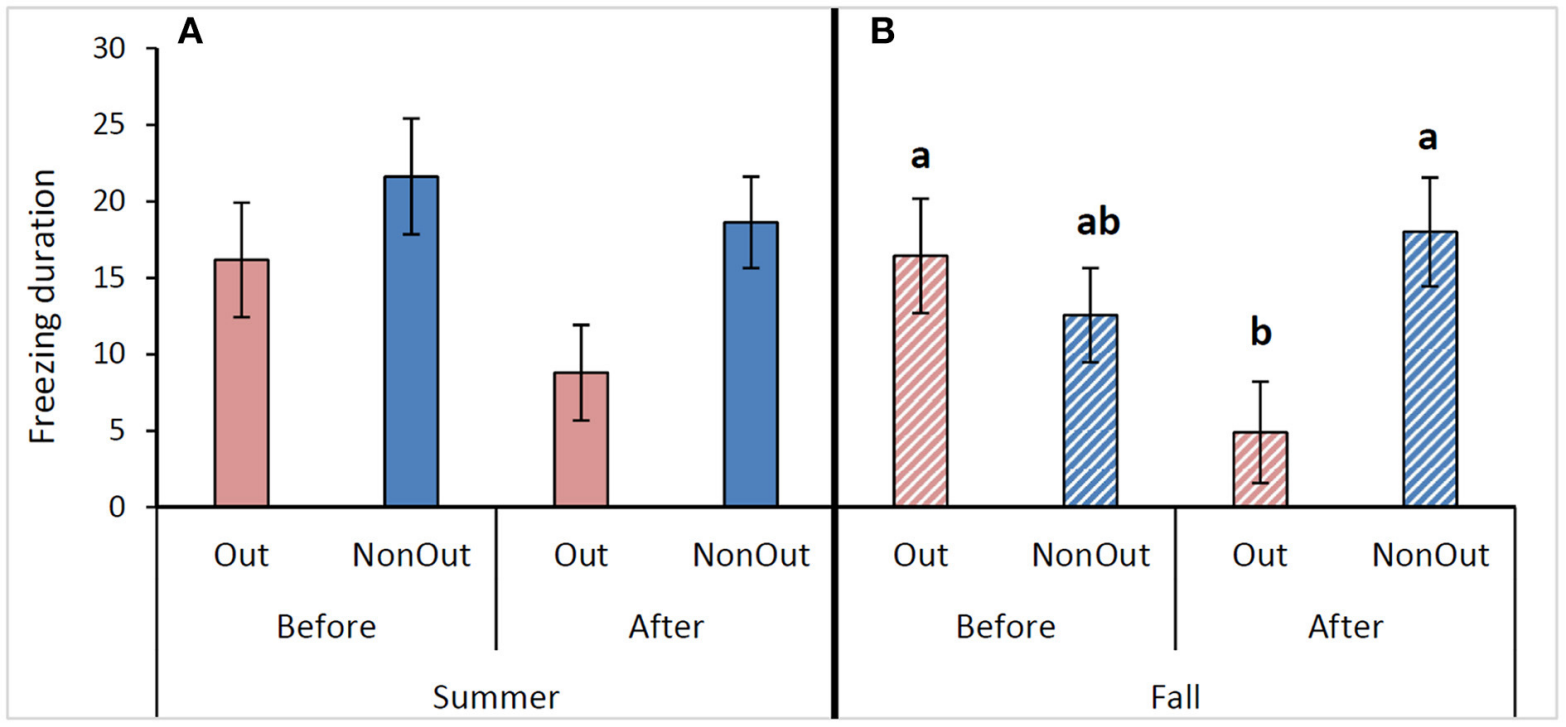
Mean (± S.E.) of freezing duration (s) for the suddenness test, measured after dropping the object in front of the cows. Values are displayed before and after an outside exercise period (Out) vs. remaining in tie-stalls without an exercise period (NonOut), for the summer **(A)** and fall seasons **(B)**. Means with different letters are significantly different (*P* < 0.05).

In summer, the time spent freezing tended to vary depending on the phase (*F*_1,29_ = 3.73; *P* = 0.06): after the outing period, cows tended to spent less time freezing. There was also a tendency for treatment effect (*F*_1,29_ = 3.59; *P* = 0.07): Out cows spent less time freezing than NonOut cows (Figure 2A). There were no significant difference according to treatment^*^phase (*F*_3,27_ = 0.66; *P* = 0.42). In fall, the time spent freezing was different according to phase^*^treatment (*F*_3,24_ = 7.98; *P* = 0.009). After the outdoor exercise access period, Out cows spent less time freezing than before the treatment period (LSD: *P* = 0.021), and less than NonOut cows (LSD: *P* = 0.027, Figure 2B).

## Discussion

The results showed, in the summer and fall seasons, that cows provided with outdoor access showed less reactivity to the suddenness test than cows that remained in tie-stalls, without being completely reactionless. Tie-stall cows tethered permanently experience a routine environment throughout the year, since all activity is conducted at the stall. In routine events are consistent, predictable, and not very diversified. While providing outdoor access permits animals to express greater socialization and natural behavior (21, 22), it also means exposing them to initially unknown and diverse stimuli that could heed unforeseeable responses. The provisioning of various stimuli and so, enrichment, allows cows to develop a range of behaviors and reactions, and therefore, promotes the animal's capacities of adaptation (18). This allows the animal to adapt to changes in the environment and assess more quickly the potential for associated risk. They can therefore avoid remaining unnecessarily alert when a sudden stimulus presents itself. It has been demonstrated that the predictability of negative or positive events are very important for animal welfare as it promotes a sense of control (23, 24). On the other hand, too much routine and predictability may support habit formation and prevent animals from developing capacities that would enable them to cope with change. It is therefore important to know how to stimulate animals and their behavior in order to give them more control over disturbing events, which may result in an increased well-being and health. For example, it is possible to use a bell sound to announce the passage of a tractor, or the start of a transfer to the exercise area. Everything that allows the animal to anticipate what will happen will prepare the animal for the event.

Our study shows that cows in the winter trial exposed to outdoor exercise access had a more positive human approach score, especially in the final stages of approach where the human was in closer proximity. As our scoring is done in four steps, it could possibly induce a slight bias. Indeed, the score of a step can be influenced by the score of the previous step: if an animal was already at the end of its stall, it could hardly move further back. However, it is highly likely that the animal will still show backward movement even if it cannot finish it (i.e., do not exit the stall). Thus, this bias would tend to slightly reduce the occurrences of extremely negative scores (scores of −3). This bias is also reduced by the fact that we have averaged the score of 3 repetitions at each step. In conclusion, we believe that the only possible effect of this bias is not to distinguish certain extreme negative cases but does not impact the positive cases. Therefore, a more positive score for cows with access to outdoor space, is probably not affected by this bias to the point of changing the direction of the interpretation of our results. This means that cows could be approached and taken by halters for routine manipulations more easily than cows that did not experience outdoor exercise access. A first potential explanation would be that cows provided with outdoor exercise access became accustomed, over the treatment period, to being regularly handled. As such, the response of fear and avoidance toward handling would have decreased as shown in other studies (25, 26). However, this effect was not present for the summer trial, since cows with outdoor exercise access had an approach score that was not different, regardless of approach stage, from cows that remained at their tie-stall. We may therefore conclude that the results were not explained by a simple habituation toward the handling process.

A second potential explanation would be that the sample group of cows observed in the summer trial were more fearful than cows observed in the winter trial, thus masking the effect of habituation to the handling process. Indeed, by reacting more to a human movement, which could take the animal by surprise, cows could show more fearful behaviors toward a human stimulus. However, we have observed that cows provided with outdoor access in the summer trial were less reactive after the treatment period than cows that remained in their stall. Thus, a simple cross effect between fear and habituation of handling/human cannot explain the results obtained. The observed effect may be directly linked to the valence of the relationship between animals and humans.

The attachment of the halter for these tie-stall cows is an event generally associated to be negative, since this occurs rarely and most often for changing stalls or medical attention. It is a disruption of daily routine, a restriction of access to resources such as feed or lying, and may potentially be associated with painful veterinary care. As part of the handling procedure for leading cows to the outdoor exercise area, halters were put on to secure the cow's removal from the stall. We could therefore conclude that cows provided with outdoor exercise access associated the halter with a predictable positive event, and subsequently, were more accepting and easily handled at the neck (the halter level). This was observed for the winter but not for the summer trial. It has already been shown that cows are sensitive to the way they are handled (11), and that aversive manipulations modify their relationship with humans (27). Cows are even able to discriminate between people based on their past experiences with them, and thus may react positively or negatively to their contact (10, 28, 29). One of the main differences between the summer and winter trials is that, in winter, the cows moved freely in the outside corridor and the handlers only intervened to push them when they stopped moving for too long during this part of the trip. In the summer, on the other hand, the cows were more excited and made many attempts to run. In order to avoid injuries to both the cows and humans, handlers were also placed in front of the cows to regularly calm them and stop them from running with halter. In the fall, there were someone in the front to help to stop the cows when running, but were more inclined to let the cows move faster/run a little in some occasions if no possible danger was assessed. In the summer, cows were more regularly caught at the halter to restrict their movements. Presumably, their movement was more restricted, and so the handling could have been perceived in a more negative way compared to cows who moved freely in the winter trial. We cannot assume that cows perceived the handling aversively, since they did not show a more negative approach score than the control cows, but they likely did not perceive the experience in a positive way. We thus conclude that cows provided with outdoor exercise access during the winter trial have an altered and more positive perception of the halter placement, while the summer Out cows perceived the halter more negatively. It should be emphasized that the motivation of cows to go outdoors was very apparent in both the summer and winter trials (Aigueperse et al. in prep), but the association of a negative event, particularly a strong one, will often impact behavior more heavily than a positive event (28, 30).

In conclusion, our study shows that the provision of regular outdoor access to tie-stall cows may therefore reduce reactivity under certain conditions. If we assume that this was an effect of providing stimuli linked to the varied conditions of the external environment, the exact mechanisms involved in changes to reactivity require further investigation. In addition, we showed that cow handling during these outings also had an impact on the cow's relationship with humans, and therefore on their future ease of handling. The provisioning of regular exercise for tie-stall cows is seen as a source of enrichment and improvement of quality of life in animals (31–33). To demonstrate the positive effect of this practice, many factors must be considered, including its impact on the health and locomotor capacities of the animals, how enrichment is provided (i.e., type of access, space, etc.), but also the handling of animals during the process. All of these factors may affect the behavior of cows, and their perception of the experience and so, have an impact on their well-being. Proper handling taking in consideration the cow's reactivity and experience gained from multiple handling sessions can improve the behavior and perception of animals. In addition, it can improve the well-being of the handler through more safety, a better relationship with their animal and a better vision of their work.

## Data Availability Statement

The raw data supporting the conclusions of this article will be made available by the authors, without undue reservation.

## Ethics Statement

The animal study was reviewed and approved by Animal Care Committee of McGill University and affiliated hospitals and research institutes (protocol #2016-7794).

## Author Contributions

NA and EV designed the study. NA performed the experiments and analyzed the data. NA wrote the original draft of the article and all the author finalized the final version. EV provided funding. All authors contributed to the article and approved the submitted version.

## Conflict of Interest

The authors declare that the research was conducted in the absence of any commercial or financial relationships that could be construed as a potential conflict of interest.
