# Supplementary material for: Providing an Outdoor Exercise Area Affects Tie-Stall Cow Reactivity and Human-Cow Relations
Source: Front Vet Sci. 2021 Jan 12;7:597607. doi: 10.3389/fvets.2020.597607 (PMC7835404; doi:10.3389/fvets.2020.597607)
Supplement: Supplementary file 3 [file Data_Sheet_1.docx]

Supplementary Material

# Standard Operating Procedure for leading cow to the exercise area

This procedure was followed by the handlers, when leading the cows to the outdoor exercise area. There were specific steps to help to move the cows forward and, when the season needed it, to slow the cows down.

*Inside the farm building (way out)*: Farm staff will lead the cows outside by their halter. One person will first unclip the chain, then another person will lead the cow out by the halter, and release her once outside, at the exit of the farm building. This is repeated for each cow, one by one.

*Outside (way out and way in)*: Once the 2 (or 3 in fall) cows from each block are released at the exit of the farm building, in the alley leading to the exercise areas, 2 handlers will follow the group to the exercise yard. Cows must be given the opportunity to walk on their own. Handlers will not encourage cows to move forward, unless the cows stop more than 10s.

1. Steps to encourage cow to walk forward were:
   1. **With no contact with the cow**: talking to cows with calm, soft voice or in conversational tone, including quiet whistling; or waving arms/clapping hands. Wait 10 seconds. If cow does not react, pass to the next step.
   2. **With soft contact with the cow**: tap or poke rump with hand or tap dewclaw with foot. Wait 10 seconds. If cow does not react, pass to the next step.
   3. **With moderate rougher contact**: using halter, move cow 2 steps forward, then let go. If no movement, wait 10 seconds and repeat.
2. A third handler is placed in front of the cows, at a distance of at least 1 cow. If cows are very excited and trying to run or gallop, this handler can intervene to calm down the cows. Steps to slow down cows were:
3. **With no contact with the cow:** talking to cows with calm, soft voice or in conversational tone, including quiet whistling; or waving arms/clapping hands. If cow does not react, pass to the next step.
4. **With contact:** taking the halter and keeping it to move 5 steps forward slowly, then let go but staying on the front of the cow.

*Inside (way in)*: Farm staff will lead each cow, using her halter, back to her stall inside the barn. Only one cow at a time will be taken inside.

In winter, we followed this procedure except for part (B), where the cows were free to move forward when they wished.

In summer, we followed all of these instructions, parts (A) and (B) so, when the cows moved too fast, they were severely restricted.

In the fall, the step 2 of part (B) was not applied: the third handler gently stopped the cow and then let her go.

# Supplementary Tables

**Table S2**: Linear Mixed Model for scores of human test: effect of phase and treatment in summer, winter and fall.

| Effect | Estimate | SE | CI_lower_ | CI_upper_ | t | P | F | | df | | P | |
| --- | --- | --- | --- | --- | --- | --- | --- | --- | --- | --- | --- | --- |
| **SUMMER** |  |  |  |  |  |  |  | |  | |  | |
| ***Model Stage 1*** | | | | | | | |  | |  | |  |
| (Intercept) | -0.381 | 0.228 | -0.849 | 0.086 | -1.673 | 0.106 |  | |  | |  | |
| PHASE |  |  |  |  |  |  | 1.546 | | 29 | | 0.224 | |
| Before (reference) |  |  |  |  |  |  |  | |  | |  | |
| After | -0.007 | 0.367 | -0.76 | 0.746 | -0.018 | 0.986 |  | |  | |  | |
| TREATMENT |  |  |  |  |  |  | 0.002 | | 29 | | 0.968 | |
| Out (reference) |  |  |  |  |  |  |  | |  | |  | |
| NonOut | 0.298 | 0.322 | -0.364 | 0.959 | 0.923 | 0.364 |  | |  | |  | |
| PHASE*TREATMENT |  |  |  |  |  |  | 1.481 | | 27 | | 0.234 | |
| After*NonOut | -0.617 | 0.507 | -1.658 | 0.423 | -1.217 | 0.234 |  | |  | |  | |
| ***Model Stage 2*** | | | | | | | |  | |  | |  |
| (Intercept) | -0.491 | 0.326 | -1.159 | 0.177 | -1.508 | 0.143 |  | |  | |  | |
| PHASE |  |  |  |  |  |  | 0.004 | | 29 | | 0.952 | |
| Before (reference) |  |  |  |  |  |  |  | |  | |  | |
| After | 0.456 | 0.496 | -0.562 | 1.474 | 0.92 | 0.366 |  | |  | |  | |
| TREATMENT |  |  |  |  |  |  | 0.152 | | 29 | | 0.7 | |
| Out (reference) |  |  |  |  |  |  |  | |  | |  | |
| NonOut | 0.281 | 0.463 | -0.669 | 1.231 | 0.607 | 0.549 |  | |  | |  | |
| PHASE*TREATMENT |  |  |  |  |  |  | 2.014 | | 27 | | 0.167 | |
| After*NonOut | -0.954 | 0.672 | -2.333 | 0.425 | -1.419 | 0.167 |  | |  | |  | |
| ***Model Stage 3*** | | | | | | | |  | |  | |  |
| (Intercept) | -0.066 | 0.305 | -0.691 | 0.56 | -0.215 | 0.831 |  | |  | |  | |
| PHASE |  |  |  |  |  |  | 1.964 | | 29 | | 0.173 | |
| Before (reference) |  |  |  |  |  |  |  | |  | |  | |
| After | -0.656 | 0.456 | -1.591 | 0.279 | -1.439 | 0.162 |  | |  | |  | |
| TREATMENT |  |  |  |  |  |  | 0.033 | | 29 | | 0.857 | |
| Out (reference) |  |  |  |  |  |  |  | |  | |  | |
| NonOut | -0.309 | 0.434 | -1.199 | 0.58 | -0.714 | 0.482 |  | |  | |  | |
| PHASE*TREATMENT |  |  |  |  |  |  | 0.525 | | 27 | | 0.475 | |
| After*NonOut | 0.447 | 0.617 | -0.819 | 1.713 | 0.725 | 0.475 |  | |  | |  | |
| ***Model Stage 4*** | | | | | | | |  | |  | |  |
| (Intercept) | -1.875 | 0.307 | -2.505 | -1.245 | -6.107 | 0 |  | |  | |  | |
| PHASE |  |  |  |  |  |  | 1.067 | | 29 | | 0.311 | |
| Before (reference) |  |  |  |  |  |  |  | |  | |  | |
| After | 0.779 | 0.545 | -0.339 | 1.898 | 1.429 | 0.164 |  | |  | |  | |
| TREATMENT |  |  |  |  |  |  | 0.137 | | 29 | | 0.714 | |
| Out (reference) |  |  |  |  |  |  |  | |  | |  | |
| NonOut | 0.25 | 0.434 | -0.641 | 1.141 | 0.576 | 0.57 |  | |  | |  | |
| PHASE*TREATMENT |  |  |  |  |  |  | 1.067 | | 27 | | 0.311 | |
| After*NonOut | -0.779 | 0.754 | -2.327 | 0.769 | -1.033 | 0.311 |  | |  | |  | |
|  |  |  |  |  |  |  |  | |  | |  | |
| **WINTER** |  |  |  |  |  |  |  | |  | |  | |
| ***Model Stage 1*** | | | | | | | |  | |  | |  |
| (Intercept) | -0.232 | 0.323 | -0.895 | 0.432 | -0.717 | 0.48 |  | |  | |  | |
| PHASE |  |  |  |  |  |  | 0.168 | | 27 | | 0.685 | |
| Before (reference) |  |  |  |  |  |  |  | |  | |  | |
| After | 0.303 | 0.335 | -0.386 | 0.991 | 0.904 | 0.374 |  | |  | |  | |
| TREATMENT |  |  |  |  |  |  | 0.01 | | 27 | | 0.922 | |
| Out (reference) |  |  |  |  |  |  |  | |  | |  | |
| NonOut | 0.448 | 0.424 | -0.423 | 1.319 | 1.056 | 0.3 |  | |  | |  | |
| PHASE*TREATMENT |  |  |  |  |  |  | 2.785 | | 25 | | 0.107 | |
| After*NonOut | -0.803 | 0.481 | -1.792 | 0.186 | -1.669 | 0.107 |  | |  | |  | |
| ***Model Stage 2*** | | | | | | | |  | |  | |  |
| (Intercept) | -0.374 | 0.552 | -1.509 | 0.762 | -0.676 | 0.505 |  | |  | |  | |
| PHASE |  |  |  |  |  |  | 1.477 | | 28 | | 0.235 | |
| Before (reference) |  |  |  |  |  |  |  | |  | |  | |
| After | 0.709 | 0.454 | -0.224 | 1.642 | 1.561 | 0.131 |  | |  | |  | |
| TREATMENT |  |  |  |  |  |  | 0.154 | | 28 | | 0.697 | |
| Out (reference) |  |  |  |  |  |  |  | |  | |  | |
| NonOut | 0.566 | 0.724 | -0.921 | 2.054 | 0.782 | 0.441 |  | |  | |  | |
| PHASE*TREATMENT |  |  |  |  |  |  | 1.099 | | 26 | | 0.304 | |
| After*NonOut | -0.656 | 0.626 | -1.943 | 0.631 | -1.048 | 0.304 |  | |  | |  | |
| ***Model Stage 3*** | | | | | | | |  | |  | |  |
| (Intercept) | 0.147 | 0.497 | -0.874 | 1.168 | 0.296 | 0.77 |  | |  | |  | |
| PHASE |  |  |  |  |  |  | 0.12 | | 28 | | 0.732 | |
| Before (reference) |  |  |  |  |  |  |  | |  | |  | |
| After | 0.687 | 0.418 | -0.173 | 1.547 | 1.642 | 0.113 |  | |  | |  | |
| TREATMENT |  |  |  |  |  |  | 0.429 | | 28 | | 0.518 | |
| Out (reference) |  |  |  |  |  |  |  | |  | |  | |
| NonOut | 0.409 | 0.651 | -0.93 | 1.748 | 0.628 | 0.536 |  | |  | |  | |
| **PHASE*TREATMENT** |  |  |  |  |  |  | 7.282 | | 26 | | **0.012** | |
| **After*NonOut** | -1.576 | 0.584 | -2.776 | -0.376 | -2.699 | **0.012** |  | |  | |  | |
| ***Model Stage 4*** | | | | | | | |  | |  | |  |
| (Intercept) | -1.214 | 0.598 | -2.443 | 0.015 | -2.03 | ***0.053*** |  | |  | |  | |
| PHASE |  |  |  |  |  |  | 1.968 | | 28 | | 0.173 | |
| Before (reference) |  |  |  |  |  |  |  | |  | |  | |
| **After** | 1.423 | 0.457 | 0.484 | 2.361 | 3.115 | **0.04** |  | |  | |  | |
| TREATMENT |  |  |  |  |  |  | 2.4 | | 28 | | 0.133 | |
| Out (reference) |  |  |  |  |  |  |  | |  | |  | |
| NonOut | -0.003 | 0.785 | -1.617 | 1.611 | -0.004 | 0.997 |  | |  | |  | |
| **PHASE*TREATMENT** |  |  |  |  |  |  | 9.934 | | 26 | | **0.004** | |
| **After*NonOut** | -1.969 | 0.625 | -3.253 | -0.685 | -3.152 | **0.004** |  | |  | |  | |
|  |  |  |  |  |  |  |  | |  | |  | |
| **FALL** |  |  |  |  |  |  |  | |  | |  | |
| ***Model Stage 1*** | | | | | | | |  | |  | |  |
| (Intercept) | -0.333 | 0.319 | -0.991 | 0.325 | -1.0465 | 0.306 |  | |  | |  | |
| PHASE |  |  |  |  |  |  | 1.141 | | 26 | | 0.296 | |
| Before (reference) |  |  |  |  |  |  |  | |  | |  | |
| After | 0.4 | 0.356 | -0.334 | 1.134 | 1.124 | 0.272 |  | |  | |  | |
| TREATMENT |  |  |  |  |  |  | 0.157 | | 26 | | 0.695 | |
| Out (reference) |  |  |  |  |  |  |  | |  | |  | |
| NonOut | 0.296 | 0.398 | -0.524 | 1.117 | 0.745 | 0.463 |  | |  | |  | |
| PHASE*TREATMENT |  |  |  |  |  |  | 0.539 | | 24 | | 0.47 | |
| After*NonOut | -0.326 | 0.444 | -1.242 | 0.59 | -0.734 | 0.47 |  | |  | |  | |
| ***Model Stage 2*** | | | | | | | |  | |  | |  |
| (Intercept) | 0.267 | 0.566 | -0.901 | 1.434 | 0.471 | 0.642 |  | |  | |  | |
| PHASE |  |  |  |  |  |  | 0.111 | | 26 | | 0.742 | |
| Before (reference) |  |  |  |  |  |  |  | |  | |  | |
| After | 0.133 | 0.5 | -0.898 | 1.164 | 0.267 | 0.792 |  | |  | |  | |
| TREATMENT |  |  |  |  |  |  | 0.097 | | 26 | | 0.758 | |
| Out (reference) |  |  |  |  |  |  |  | |  | |  | |
| NonOut | -0.156 | 0.706 | -1.612 | 1.301 | -0.22 | 0.827 |  | |  | |  | |
| PHASE*TREATMENT |  |  |  |  |  |  | 0.009 | | 24 | | 0.925 | |
| After*NonOut | -0.059 | 0.623 | -1.345 | 1.227 | -0.095 | 0.925 |  | |  | |  | |
| ***Model Stage 3*** | | | | | | | |  | |  | |  |
| (Intercept) | -1.067 | 0.364 | -1.819 | -0.315 | -2.928 | **0.007** |  | |  | |  | |
| **PHASE** |  |  |  |  |  |  | 10.223 | | 24 | | **0.004** | |
| Before (reference) |  |  |  |  |  |  |  | |  | |  | |
| **After** | 1.4 | 0.481 | 0.407 | 2.393 | 2.911 | **0.008** |  | |  | |  | |
| TREATMENT |  |  |  |  |  |  | 0 | | 24 | | 0.993 | |
| Out (reference) |  |  |  |  |  |  |  | |  | |  | |
| NonOut | 0.437 | 0.454 | -0.501 | 1.375 | 0.962 | 0.346 |  | |  | |  | |
| PHASE*TREATMENT |  |  |  |  |  |  | 2.161 | | 24 | | 0.155 | |
| After*NonOut | -0.882 | 0.6 | -2.12 | 0.356 | -1.47 | 0.155 |  | |  | |  | |
| ***Model Stage 4*** | | | | | | | |  | |  | |  |
| (Intercept) | -1.667 | 0.568 | -2.839 | -0.494 | -2.934 | **0.007** |  | |  | |  | |
| **PHASE** |  |  |  |  |  |  | 12.139 | | 26 | | **0.002** | |
| Before (reference) |  |  |  |  |  |  |  | |  | |  | |
| **After** | 1.4 | 0.407 | 0.559 | 2.241 | 3.436 | **0.002** |  | |  | |  | |
| TREATMENT |  |  |  |  |  |  | 0 | | 26 | | 0.995 | |
| Out (reference) |  |  |  |  |  |  |  | |  | |  | |
| NonOut | 0.519 | 0.708 | -0.944 | 1.981 | 0.732 | 0.471 |  | |  | |  | |
| ***PHASE*TREATMENT*** |  |  |  |  |  |  | 4.101 | | 24 | | ***0.054*** | |
| ***After*NonOut*** | -1.029 | 0.508 | -2.078 | 0.02 | -2.025 | ***0.054*** |  | |  | |  | |

Statistically significant results appear in bold, tendencies in bolt italic. CI: confidence limit in 95%. Animal id nested in pair is random effect.

**Table S2**: Linear Mixed Model for Time spent Freezing: effect of phase and treatment in summer and fall suddenness tests.

| Effect | Estimate | SE | CI_lower_ | CI_upper_ | t | P | F | df | P |
| --- | --- | --- | --- | --- | --- | --- | --- | --- | --- |
| **SUMMER** |  |  |  |  |  |  |  |  |  |
| (Intercept) | 16.173 | 3.745 | 8.488 | 23.858 | 4.318 | 0 |  |  |  |
| ***PHASE*** |  |  |  |  |  |  | 3.725 | 29 | ***0.064*** |
| Before (reference) |  |  |  |  |  |  |  |  |  |
| ***After*** | -7.379 | 3.926 | -15.435 | 0.677 | -1.879 | ***0.071*** |  |  |  |
| ***TREATMENT*** |  |  |  |  |  |  | 3.594 | 29 | ***0.069*** |
| Out (reference) |  |  |  |  |  |  |  |  |  |
| NonOut | 5.452 | 5.324 | -5.471 | 16.375 | 1.024 | 0.315 |  |  |  |
| PHASE*TREATMENT |  |  |  |  |  |  | 0.663 | 27 | 0.423 |
| After*NonOut | 4.379 | 5.378 | -5.471 | 16.375 | 0.814 | 0.423 |  |  |  |
| **FALL** |  |  |  |  |  |  |  |  |  |
| (Intercept) | 16.44 | 3.739 | 8.753 | 24.126 | 4.396 | 0 |  |  |  |
| PHASE |  |  |  |  |  |  | 1.029 | 28 | 0.32 |
| Before (reference) |  |  |  |  |  |  |  |  |  |
| **After** | -11.544 | 4.716 | -21.238 | -1.85 | -2.448 | **0.021** |  |  |  |
| TREATMENT |  |  |  |  |  |  | 1.16 | 28 | 0.291 |
| Out (reference) |  |  |  |  |  |  |  |  |  |
| NonOut | -3.884 | 4.847 | -13.847 | 6.079 | -0.801 | 0.43 |  |  |  |
| **PHASE*TREATMENT** |  |  |  |  |  |  | 7.982 | 26 | **0.009** |
| **After*NonOut** | 16.988 | 6.013 | 4.628 | 29.348 | 2.825 | **0.009** |  |  |  |

Statistically significant results appear in bold, tendencies in bolt italic. CI: confidence limit in 95%. Animal id nested in pair is random effect.

**Table S3**: Cumulative Link Mixed Model for Reaction Score: effect of phase and treatment in summer and fall suddenness tests.

| Effect | Estimate | SE | CI_lower_ | CI_upper_ | t | P | F | df | P |
| --- | --- | --- | --- | --- | --- | --- | --- | --- | --- |
| **SUMMER** |  |  |  |  |  |  |  |  |  |
| Treshold |  |  |  |  |  |  |  |  |  |
| 0 -> 1 | -2.536 | 0.931 | -4.453 | -0.62 | -2.725 | **0.012** |  |  |  |
| 1 -> 2 | -1.139 | 0.743 | -2.668 | 0.391 | -1.533 | 0.138 |  |  |  |
| 2 -> 3 | 1.514 | 0.786 | -0.105 | 3.134 | 1.926 | ***0.066*** |  |  |  |
| PHASE |  |  |  |  |  |  | 0.139 | 27 | 0.713 |
| Before (reference) |  |  |  |  |  |  |  |  |  |
| After | -1.558 | 1.022 | -3.663 | 0.546 | -1.525 | 0.14 |  |  |  |
| **TREATMENT** |  |  |  |  |  |  | 7.648 | 27 | **0.011** |
| Out (reference) |  |  |  |  |  |  |  |  |  |
| NonOut | 0.952 | 0.995 | -1.096 | 3.001 | 0.958 | 0.347 |  |  |  |
| ***PHASE*TREATMENT*** |  |  |  |  |  |  | 3.211 | 25 | ***0.085*** |
| ***After*NonOut*** | 2.592 | 1.447 | -0.387 | 5.572 | 1.792 | ***0.085*** |  |  |  |
| **FALL** |  |  |  |  |  |  |  |  |  |
| Treshold |  |  |  |  |  |  |  |  |  |
| 0 -> 1 | -4.501 | 1.252 | -7.085 | -1.918 | -3.596 | **0.001** |  |  |  |
| 1 -> 2 | -1.337 | 0.825 | -3.04 | 0.367 | -1.62 | 0.118 |  |  |  |
| 2 -> 3 | 0.675 | 0.777 | -1.229 | 1.978 | 0.482 | 0.634 |  |  |  |
| **PHASE** |  |  |  |  |  |  | 4.591 | 26 | **0.044** |
| Before (reference) |  |  |  |  |  |  |  |  |  |
| **After** | -3.383 | 1.332 | -6.131 | -0.634 | -2.54 | **0.018** |  |  |  |
| TREATMENT |  |  |  |  |  |  | 0.815 | 26 | 0.376 |
| Out (reference) |  |  |  |  |  |  |  |  |  |
| NonOut | -0.996 | 0.992 | -3.043 | 1.051 | -1.004 | 0.325 |  |  |  |
| **PHASE*TREATMENT** |  |  |  |  |  |  | 4.519 | 24 | **0.044** |
| **After*NonOut** | 3.383 | 1.591 | 0.098 | 6.667 | 2.126 | **0.044** |  |  |  |

Statistically significant results appear in bold, tendencies in bolt italic. CI: confidence limit in 95%. Animal id nested in pair is random effect.

# Supplementary Figures

**VIDEO S1 |** Human reactivity test. The test person have approached the test cow every 5 s according to the protocol sequence: (1) 1 step, arms placed alongside the body; (2) 1 step, arms placed alongside the body; (3) One arm stretched out at approximately 45 °; (4) Outstretched hand placed on the chain at the base of the neck. For each step cow’s reaction is scored between -3/+3. Here the scores are: stage 1= -2; stage 2=-1; stage 3= -1; stage 4= -2.

**VIDEO S2 |** Sudden test. A manipulator, remained approximately two stalls away from the target cow, dropped the keel by releasing the string suddenly and the cow were filmed for 30 s. The time in freezing (here 12 s) and a score of reaction (here 2) were determined to each cow.
